# Enhanced secretion of human α1-antitrypsin expressed with a novel glycosylation module in tobacco BY-2 cell culture

**DOI:** 10.1080/21655979.2019.1604037

**Published:** 2019-04-17

**Authors:** Ningning Zhang, Tristen Wright, Paige Caraway, Jianfeng Xu

**Affiliations:** a Arkansas Biosciences Institute, Jonesboro, AR, USA; b Department of Biological Sciences, Jonesboro, AR, USA; c College of Agriculture, Arkansas State University, Jonesboro, AR, USA

**Keywords:** Plant cell culture, human α1-antitrypsin, recombinant protein, hydroxyproline-*O*-glycosylation, secretion

## Abstract

Expression of recombinant proteins fused to a novel glycomodule tag, termed hydroxyproline (Hyp)-*O*-glycosylated peptides (HypGP), was earlier found to boost secreted protein yields up to 500-fold in plant cell culture. Here, this technology was applied to the expression of human protease inhibitor α1-antitrypsin (AAT) in tobacco BY-2 cell culture. A designer HypGP tag composed of a ‘Ala-Pro’ motif of 20 units, or (AP)_20_, was engineered either at the N- or C-terminal end of AAT. The (AP)_20_ tag substantially increased the secreted yields of the recombinant AAT up to 34.7 mg/L. However, the (AP)_20_-tagged AAT products were frequently subjected to proteolytic processing. The intact AAT-(AP)_20_ along with some of the truncated AAT domains exhibited desired biological activity in inhibiting elastase. The results from this research demonstrated that the designer (AP)_20_ module engineered in BY-2 cells could function as a molecular carrier to substantially enhance the secreted yields of the recombinant AAT.

## Graphical Abstract

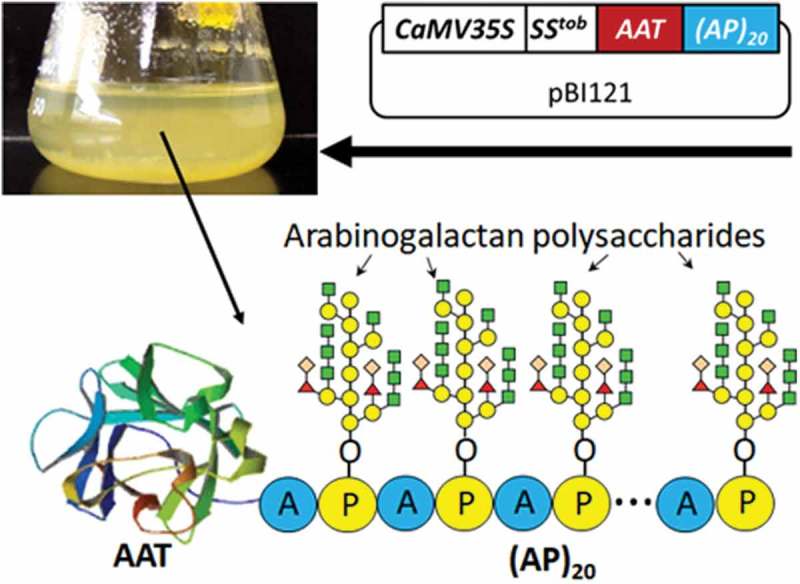

## Introduction

Human α1-antitrypsin (AAT) is a serine protease inhibitor (serpin) that functions in maintaining appropriate levels of neutrophil elastase in the respiratory system and other proteinases in the circulatory system [1]. Functional AAT binds irreversibly to neutrophil elastase in the lungs, protecting the lung tissue from being destroyed. A hereditary deficiency of AAT accounts for more than 1% of all chronic obstructive pulmonary cases and young persons with emphysema [2]. Those suffering from AAT deficiency can undergo replacement therapies with plasma-derived AAT to lessen the effects. In addition, AAT has also shown promise for the treatment of respiratory conditions related to cystic fibrosis, another genetic orphan disease characterized by elevated levels of elastase in sputum. However, problems arise with these treatments due to high demand for plasma donors, prohibitive cost to the patient at $100,000 per year [3], risk of unknown blood-borne pathogens/diseases, and the frequency of treatment at least once a week [3]. Thus, there is a pressing need for safer, lower cost and expandable sources of AAT.

Recombinant AAT has been produced in a number of expression systems, including *E. coli* [4], yeast [5], mammalian cell [6], and whole-plants such as *Nicotiana benthamiana* [7], tomato [8] and tobacco chloroplasts [9]. However, there have often been challenges associated with cost, safety, bioactivity and authenticity for each expression system. Plant cell suspension cultures are emerging as a promising alternative bioproduction system for recombinant pharmaceuticals as it integrates the merits of whole plant cultivation, microbial fermentation and mammalian cell culture [10,11]. Particularly, as eukaryotic organisms plant cells can produce complex proteins with correct post-translational modifications (e.g., glycosylation) without risk of contamination by human pathogens [12,13]. In fact, recombinant AAT has earlier been produced using plant cell culture, mainly in rice cells using an inducible α-amylase promoter–*RAmy3D* (induced by sugar starvation) [14–16], which generated a remarkably high secreted AAT yield of 247 mg/L [17]. However, the growth rates, characteristics, and stability of rice cell lines cannot compare with those of tobacco bright yellow-2 (BY-2) cell, and the viability of rice cell is substantially decreased when cultivated in a sucrose-starvation medium to induce gene expression [11]. Tobacco BY-2 cell line is extremely attractive as a bioproduction system due to its fast growth rate (doubling time as short as 11 hr) and ease of genetic transformation and cell cycle synchronization [18]. However, low protein productivity has been the bottleneck limiting the commercial application of the BY-2 cell culture system.

Engineering novel glycomodules composed of a hydroxyproline (Hyp)-*O*-glycosylated peptide (HypGP) was earlier found to dramatically increase the secreted yields of fused proteins as much as 500-fold in plant cell culture [19–21]. The HypGP is made of a proline-rich peptide backbone (e.g., tandem repeats of a dipeptide ‘Ser-Pro’ motif) subjected to intensive post-translational modifications in plant cells, including proline hydroxylation and subsequent Hyp-*O*-glycosylation with arabinogalactan polysaccharides [22,23]. Our previous studies indicated that the glycosylated HypGP module could function as a ‘molecular carrier’ to boost the secretion of fused proteins, including interferon α2b, human growth hormone and green fluorescent protein, presumably by facilitating efficient transport of the proteins into extracellular space and preventing proteolytic degradation of the proteins [20,21,24]. Most recently, the HypGP engineering technology was extended to green microalgae (*Chlamydomonas reinhardtii*), where engineering (SP)_n_ tags (n = 10, 20) enhanced the secreted yields of a fused protein by up to 12-fold [25].

Inspired by early success, we continued to apply this technology to producing AAT, a relatively large-sized protein (52 kDa) compared with those in our previous studies (18 to 27 kDa). Furthermore, a different HypGP design comprised of a ‘Ala-Pro’ motif of 20 units, or (AP)_20_ was used in this study. Early studies indicated that both the ‘Ser-Pro’ and ‘Ala-Pro’ motifs were 100% hydroxylated and underwent 100% polysaccharide addition in plant cells [26,27]. However, compared with the (SP)_n_ motifs, the (AP)_20_ lacks the amino acid Ser that might give rise to *O*-glycosylation [28,29], thus generating more homogenous Hyp-*O*-glycans. In this report, AAT was expressed in BY-2 cell with a (AP)_20_ tag, aiming to achieve high secreted protein yields. The Hyp-O-glycosylation, secreted protein yields, and biologic activity of the (AP)_20_-tagged AAT were characterized.

## Materials and methods

### Construction of expression vectors and transformation of BY-2 cell

**Figure 1.**
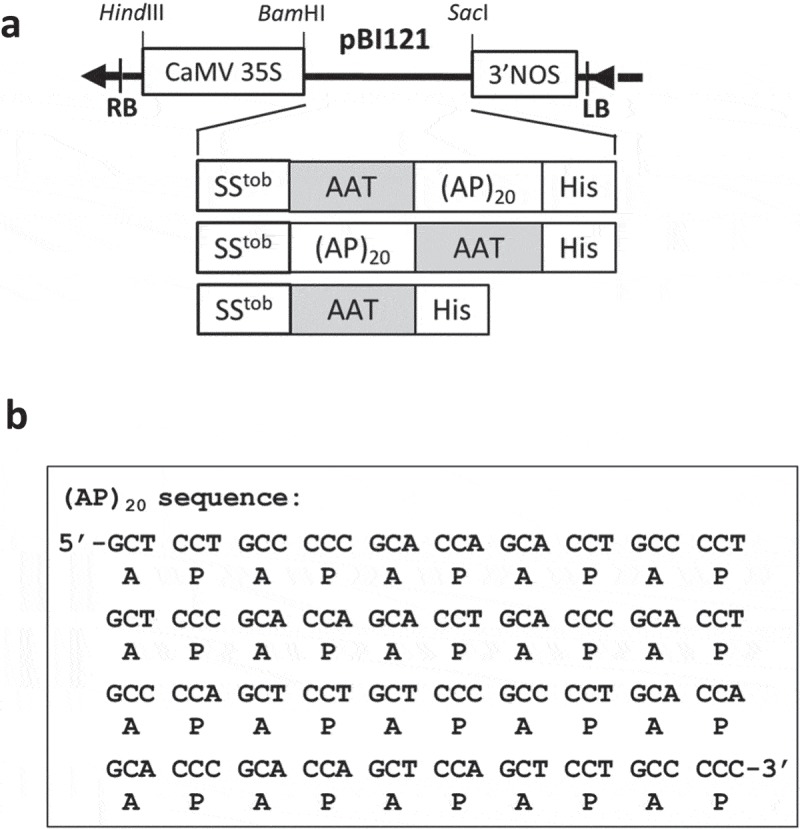
**Schematic of the gene constructions in *pBI121* expression vector (a) and the cDNA sequence of the designed synthetic (AP)_20_ gene (b)**. When the gene constructs encoding the (AP)_20_-tagged AAT are expressed in BY-2 cells, the clustered non-contiguous Pro (P) residues in the (AP)_20_ module are expected to hydroxylate to be Hyp, and subsequently *O*-glycosylated with arabinogalactan polysaccharides [22,30]. (AP)_20_: twenty tandem repeats of ‘Ala-Pro’ motif; SS^tob^: tobacco extensin signal sequence; CaMV35S: 35S cauliflower mosaic virus promoter; 3ʹNOS: nopaline synthase terminator; His: 6 × His tag.

The gene constructs encoding human AAT with a C-terminal (AP)_20_ tag, or AAT-(AP)_20_, was synthesized in *pUC18* plasmid by GenScript (Piscataway, NJ). The native *AAT* gene sequence was used in this study, and its encoded amino acid sequence is shown in Supplementary Fig. 1. The *AAT* gene fragment was then amplified by PCR using the primer pair AAT-F1 and AAT-R (Supplementary Table 1), and subcloned into the plasmid *pUC18-(AP)_20_-EGFP* at the *NcoI* and *BsrGI* sites by the In-Fusion HD Cloning Kit (Takara Bio USA, Inc) to generate *pUC18-(AP)_20_-AAT*. Similarly, the *AAT* gene was PCR amplified using the primer pair AAT-F2 and AAT-R (Supplementary Table 1), and subcloned into the plasmid *pUC18-EGFP* at the *XmaI* and *BsrGI* sites to generate *pUC18-AAT*. All the three gene constructs were subcloned into the plant expression vector *pBI121-SS^tob^-EGFP* [22,30] at the *XmaI* and *BsrGI* sites to generate *pBI121-SS^tob^-AAT, pBI121-SS^tob^-AAT-(AP)_20_* and *pBI121-SS^tob^-(AP)_20_-AAT*, respectively (Figure 1), and then stably transformed into BY-2 cell with the *Agrobacterium*-mediated approach [31]. Transgene expression was driven by the cauliflower mosaic virus 35S promoter (CaMV35S).

For verification of genome integration of the *SS^tob^-AAT, SS^tob^-AAT-(AP)_20_* and *SS^tob^-(AP)_20_-AAT* constructs in transgenic BY-2 cells, the genomic DNA was extracted from different transgenic cell lines and the target genes were amplified by PCR using the primer pair of SS-F and SS-R (Supplementary Table 1). The PCR amplicons were then separated by 1% (w/v) agarose gel electrophoresis.

### Culture of BY-2 cells and determination of cell biomass

Transgenic BY-2 cells expressing three types of AAT proteins were grown in Schenk & Hildebrandt (SH) medium [32] supplemented with 0.4 mg/L 2,4-dichlorophenoxyacetic acid (2,4-D), 0.1 mg/L kinetin and 34 g/L sucrose. Cell suspension cultures were conducted in 250 ml flasks containing 75 mL medium, which were rotated at 90 rpm on a gyratory shaker at 25°C. Subcultures were carried out every week. For kinetic studies of the cell growth and protein secretion, suspension cultured cells were collected at an interval of 2 days for determination of cell biomass and secreted AAT products. Cultured cells were harvested by vacuum filtration and washed three times with distilled water before the determination of fresh weight (FW). The cells were then dried in an oven at 70°C for 48 hr to determine dry weight (DW). Cell biomass and secreted protein yields were analyzed for each sample, as described below.

### Purification of protein from culture media

The media harvested after 10 days of BY-2 cell culture were pre-separated with 35% to 60% (w/v) ammonium sulfate precipitation. The fraction precipitated with 60% (w/v) ammonium sulfate was further purified with nickel affinity chromatography with the Ni-NTA Spin Columns (Qiagen, MA) following the manufacturer’s procedures.

### Immunoblotting analysis

For Western blotting assay, the secreted AAT products accumulated in culture media were separated by sodium dodecyl sulfate-polyacrylamide gel electrophoresis (SDS-PAGE) as described earlier [24,33]. The protein bands were then electrotransferred onto a 0.2 μm nitrocellulose membrane (Thermo Scientific Pierce, Rockford, lL), and detected using the rabbit anti-AAT polyclonal antibody (Assaypro, St Charles, MO), and the goat anti-rabbit IgG (H + L) secondary antibody, peroxidase conjugated (Jackson Immuno Research labs, West Grove, PA). Protein blots were detected using the SuperSignal® West Pico Chemiluminescent Substrate (Thermo Scientific Pierce, Rockford, lL) in accordance with the manufacturers’ procedures. For dot blotting, 1.0–1.5 μl of medium sample was heated at 90°C for 10 min before being blotted onto a 0.2 μm nitrocellulose membrane, and the AAT products were detected using the same approach as that described for the Western blotting assay.

### Monosaccharide composition assay

The monosaccharide compositions of purified AAT-(AP)_20_ fusion protein was determined at the Complex Carbohydrate Research Center, The University of Georgia (Athens, GA), as described earlier [34].

### N-terminal peptide sequencing

Purified transgene products were separated on a 10% Tris-HCl gel and stained with Coomassie blue R-250. The target bands were cut from the gel for peptide sequencing by Edman degradation at the Protein Facility of the Iowa State University (Ames, IA).

### Precipitation with (β-D-galactosyl)_3_-yariv reagent

(*β*-D-galactosyl)_3_-Yariv (Biosupplies Australia Pty Ltd, Australia) was dissolved in 2% (w/v) NaCl at the concentration of 1.0 mg/ml. (AP)_20_-tagged protein (50 and 100 µg) was dissolved in 300 µl of distilled H_2_O. An equal volume of protein sample and Yariv reagent was mixed and incubated at room temperature for 1 hr before the precipitates were pelleted in a microcentrifuge (Eppendorf MiniSpin^TM^) at full speed (13,200 rpm). The resulted pellets were washed with distilled H_2_O, dissolved in 0.1 M NaOH, and then the absorbance was measured at 420 nm. Tobacco BY-2 cell-secreted (SP)_32_-EGFP [33] was used as a positive control.

### Quantification of recombinant AAT products

Secreted AAT and AAT *equivalent* of (AP)_20_-AAT or AAT-(AP)_20_ in BY-2 cell culture media was assayed using a sandwich Human AAT ELISA kit (Assaypro, St Charles, MO). For quantification of intracellular AAT products, the proteins were first extracted from harvested cells with the SDS extraction buffer (150 mM Tris-HCl, pH 6.8, 30% glycerol, 6% SDS, 5 mM EDTA) as described by Zhang et al. [33], and then assayed by the sandwich ELISA.

### Biological activity assay of recombinant AAT products

Biological activity of BY-2 cell-secreted AAT products was assayed *in vitro* for inhibiting elastase, as described by Huang et al. [15] and McDonald et al. [17]. Briefly, 50 µl of human AAT standard (Assaypro, St Charles, MO) or recombinant AAT samples (crude media or purified protein) was added to 100 µL of assay buffer (0.15 M NaCl, 0.02 M Tris-HCl and 0.01% Tween 80, pH 8.1) in a 96-well microtiter plate. Fifty microliters of porcine pancreatic elastase (PPE, 2.8 µg/mL, Sigma, St. Louis, MO) was then added to individual wells, followed by incubation at 37°C for 15 min to initiate the inhibition of elastase. Finally, 50 µl of substrate consisting of 2 mM of N-succinyl-Ala-Ala-Ala-*p*-nitroanilide (Sigma, St. Louis, MO) was added to determine the active residual elastase that could cleave the substrate to generate chromogenic *p*-nitroanilide, which was detected at OD = 405 nm. Human AAT standard at concentrations ranging from 0 to 14 µg/mL was used to generate the calibration curve correlating the active AAT concentration to the residual PPE activity. The medium collected from wild type BY-2 cell culture was used as a negative control.

### Statistical analysis

Sample assays were carried out with three replicates, and data were presented as the mean with standard deviation (*SD*). One-way analysis of variance (ANOVA) followed by a Tukey *post hoc* range test was used to determine differences among treatments with *p* < 0.05 being significant and *P* < 0.01 extremely significant.

## Results and discussions

### Plant expression vectors were constructed

We constructed the gene constructs encoding three AAT products: one untagged control and two with a (AP)_20_ tag at either N-terminus or C-terminus, all being targeted for extracellular secretion by a signal peptide of tobacco extensins SS^tob^ [30] (Figure 1). Based on the Hyp-*O*-glycosylation ‘code’ elucidated earlier and our previous results [21,22,27,35], we predicted that all the Pro residues in the (AP)_20_ tag, upon engineered into plant cells, would undergo hydroxylation and subsequent *O*-glycosylation with arabinogalactan polysaccharides. This would greatly increase the molecular size of the recombinant AAT from ~52 kDa to more than 100 kDa.

### Engineered (AP)_20_ module enhanced extracellular secretion of recombinant AAT

**Figure 2.**
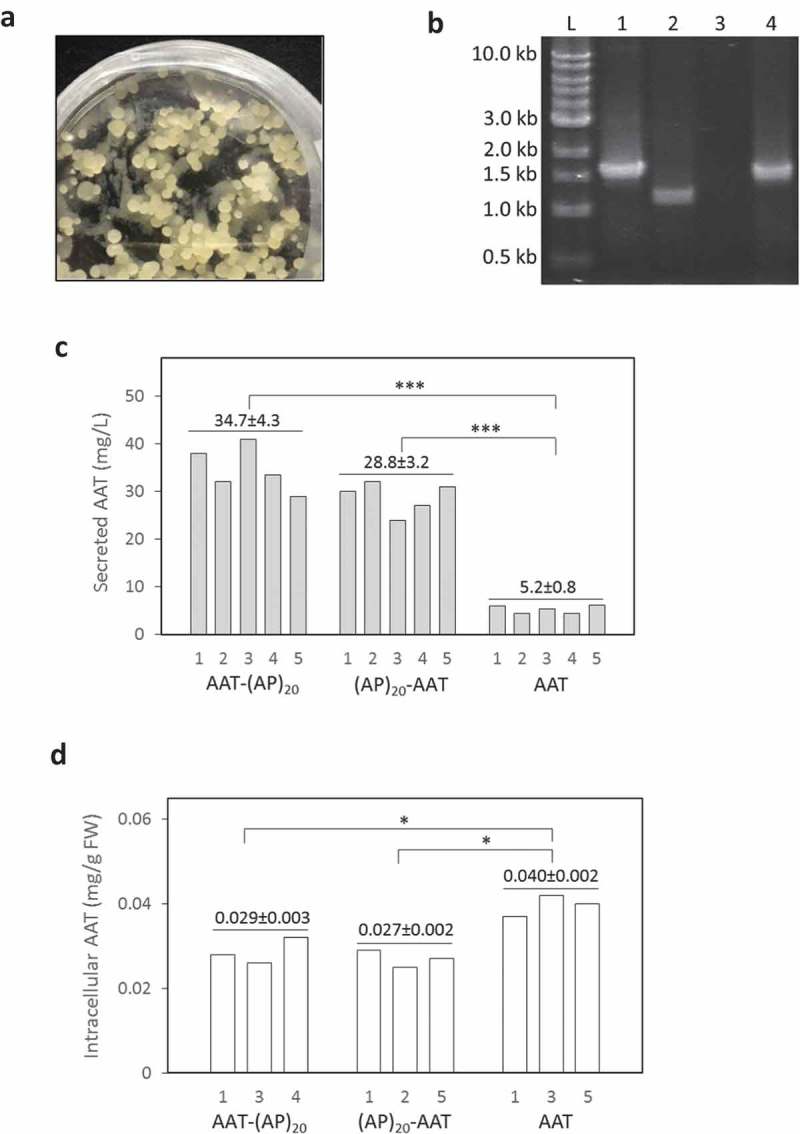
**Expression of recombinant AAT products in tobacco BY-2 cell culture.** (a) Transgenic BY-2 cell colonies appeared in a selection medium (MS medium with 100 mg/L kanamycin) 3 weeks after co-culture of wild-type BY-2 cell with *Agrobacterium*; (b) PCR detection of the target gene integration into the genome of transgenic BY-2 cell lines. L: DNA ladder, Lane 1, 2, 3, 4: PCR amplicons amplified from the genomic DNA extracted from the BY-2 cells expressing AAT-(AP)_20_, AAT control, wild type line, and (AP)_20_-AAT, respectively; (c) Secreted yields of AAT-(AP)_20_, (AP)_20_-AAT and AAT control in the transgenic BY-2 cell cultures. Five top-expression BY-2 colonies for each gene construct (# 1 to 5 on X-axis) selected by dot blotting were grown in SH medium for 10 days before the assay; (d) Intracellular AAT contents of the BY-2 cells. Three top-secretion BY-2 colonies for each construct identified in Panel (c) were assayed. Statistical significance is indicated by *(*p* < 0.05) and ***(*p* < 0.01).

With the *Agrobacterium*-mediated transformation, many stably transformed BY-2 cell colonies (calli) were obtained (Figure 2(a)). The integration of the corresponding gene construct into the genome of BY-2 calli was verified by PCR (Figure 2(b)), which showed a ~ 1.5 kb amplicon for *SS^tob^-AAT-(AP)_20_* and *SS^tob^-(AP)_20_-AAT* and a ~ 1.3 kb amplicon for *SS^tob^-AAT*. At least 30 cell lines for each gene construct were isolated and grown in liquid SH medium to screen for high-secretion lines by dot blotting assay (data not shown). Five lines for each gene construct were finally selected and subcultured for quantification of the protein yields by ELISA.

**Figure 3.**
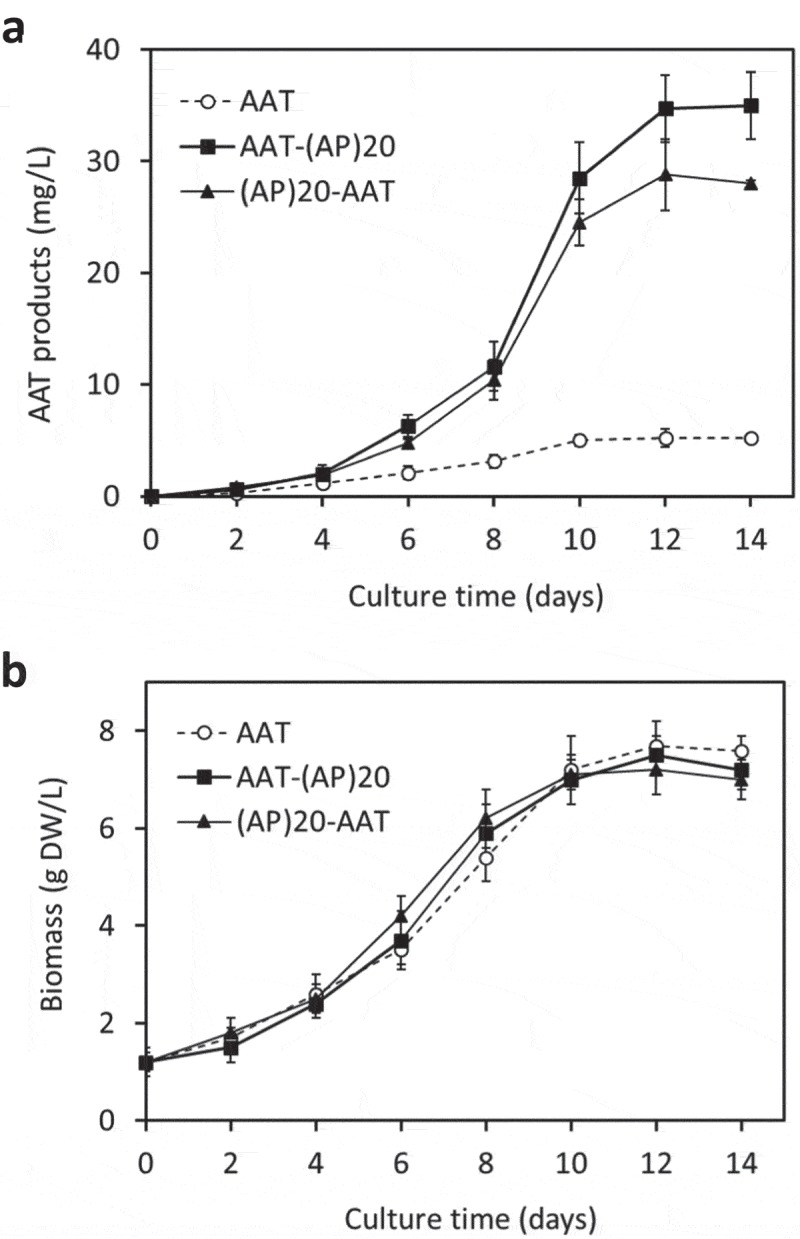
**Time course of cell growth and AAT product secretion of the transgenic BY-2 cell cultures.** (a) Secreted AAT products in the culture media; (b) Cell biomass accumulation. The error bars represent the standard deviation of three parallel cultures.

The engineered (AP)_20_ tag, either attached at the N-terminus or C-terminus of AAT, substantially enhanced the secreted yields of the recombinant protein (Figure 2(c)). The secreted AAT yields (AAT *equivalent* detected by anti-AAT ELISA) after 12 days of culture were determined to be 28.8 ± 3.2 mg/L for (AP)_20_-AAT and 34.7 ± 4.3 mg/L for AAT-(AP)_20_, which represented 5.5 to 6.7-fold greater than the expression of AAT control (5.2 ± 0.8 mg/L). This indicated that the ‘Ala-Pro’ motif-based HypGP tag design, as the (SP)_n_ tag (n = 5, 10, 20, 32) used previously [20–22,24], could function as a molecular carrier in facilitating extracellular secretion of the fused AAT protein. Time course of AAT products secretion was further investigated. As shown in Figure 3(a), dramatic accumulation of the (AP)_20_-AAT or AAT-(AP)_20_ products started after 4 days of culture until day 12, which correlated with the rapid increase in cell biomass (Figure 3(b)). The growth curves of the three genotypes of BY-2 cells were similar, all showing a growth cycle of 12 to 14 days with an exponential growth phase occurring between day 4 and 10 (Figure 3(b)). The cell biomass harvested at the end of the cultures (7.2 to 7.5 gDW/L) were comparable among the three transgenic cell lines.

When the intracellular accumulation of AAT products was examined, more AAT control (0.040 mg/gFW) than the (AP)_20_-tagged AAT (0.027 to 0.029 mg/gFW) was observed (Figure 2(d)), which was consistent with the enhanced secretion of the (AP)_20_-tagged AAT as shown in Figure 2(c). In terms of spacious distribution of the synthesized AAT products in BY-2 cell cultures, the secreted AAT with a (AP)_20_ tag accounted for 87.1% to 89.5% of the total AAT products produced (intracellular and extracellular combined). In contrast, 47.0% of AAT control was extracellularly secreted. Compared with our previous studies in which less than 5% of recombinant proteins (including EGFP, hGH, and IFNα2 that is absent of glycosylation) was usually secreted from cultured BY-2 cell [20,21,24], AAT is naturally a secreted protein in plant cell culture, where up to 247 mg/L AAT was secreted in rice cell culture [17]. This is largely attributed to AAT being a glycoprotein that carries three *N-*linked glycosylation sites (^46^Asn, ^83^Asn, and ^247^Asn) [36]. All these sites were found to be fully occupied when AAT was expressed in *N. benthamiana*, which prompted extracellular secretion of the protein [7]. In this study about half of the synthesized AAT control (47%) was secreted into BY-2 cell culture media. However, the protein secretion was substantially increased to 89.5% when engineered with the (AP)_20_ tag, leading to a 6.7-fold increase in the final secreted protein yields. This demonstrated that the HypGP engineering, specifically engineering a (AP)_20_ module in this study, was applicable for improving the secreted yield of a relatively large glycoprotein (52 kDa) in plant cell culture.

As far as we know this is the first report on AAT protein expression in tobacco BY-2 cell. However, compared with the AAT expression in rice cell with an inducible *RAmy3D* promoter, the secreted protein yield obtained in this study was still low. However, BY-2 cell has major advantages over rice cell in terms of growth rate, characteristics, and stability. Particularly, the growth rate of BY-2 cell with a doubling time as short as 11 hr [18] is much higher than rice cell whose growth doubling time was reported to be 6 to 7 days [14,16]. In addition, exchanging medium to create sucrose-starvation environments in rice cell culture to induce the gene expression (under the *RAmy3D* promoter) imposes a major technical difficulty on a large scale and advanced bioreactor culture strategies that could potentially enhance protein productivity, e.g., perfusion cultures, cannot be readily implemented [11]. The major bottleneck preventing commercial applications of the BY-2 cell culture system lies in low protein productivity, particularly low secreted protein yields. This, to a large extent, could be overcome by the HypGP engineering technology, with which secreted EGFP yields up to 168 mg/L was previously achieved [33]. Boosting protein secretion in plant cell culture by the HypGP engineering is of practical significance to the biotechnology industry, as protein products can be easily isolated from simple plant cell culture media at a substantially reduced cost compared with the protein expression in whole plants.

### Secreted AAT products were subjected to proteolytic cleavage

**Figure 4.**
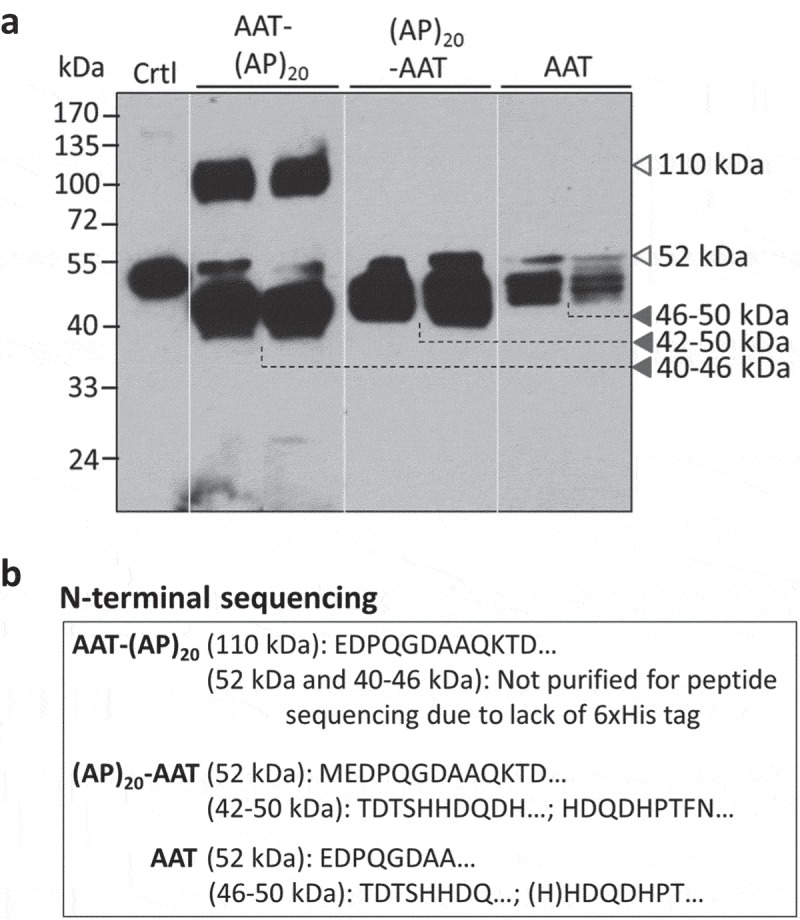
**Detection of (AP)_20_-AAT, AAT-(AP)_20_ and AAT products accumulated in BY-2 cell culture medium.** (a) Western blotting detection of the transgenic products. Two transgenic BY-2 cell lines for each gene construct were grown in liquid medium for 10 days before the assay with an anti-AAT polyclonal antibody. Crtl: AAT standard (100 ng). Sixteen microliter of culture media was loaded into each well. (b) N-terminal peptide sequencing of transgenic products. Those with a C-terminal 6 × His tag were able to be purified with the Ni-NTA Spin Columns for sequencing.

**Figure 5.**
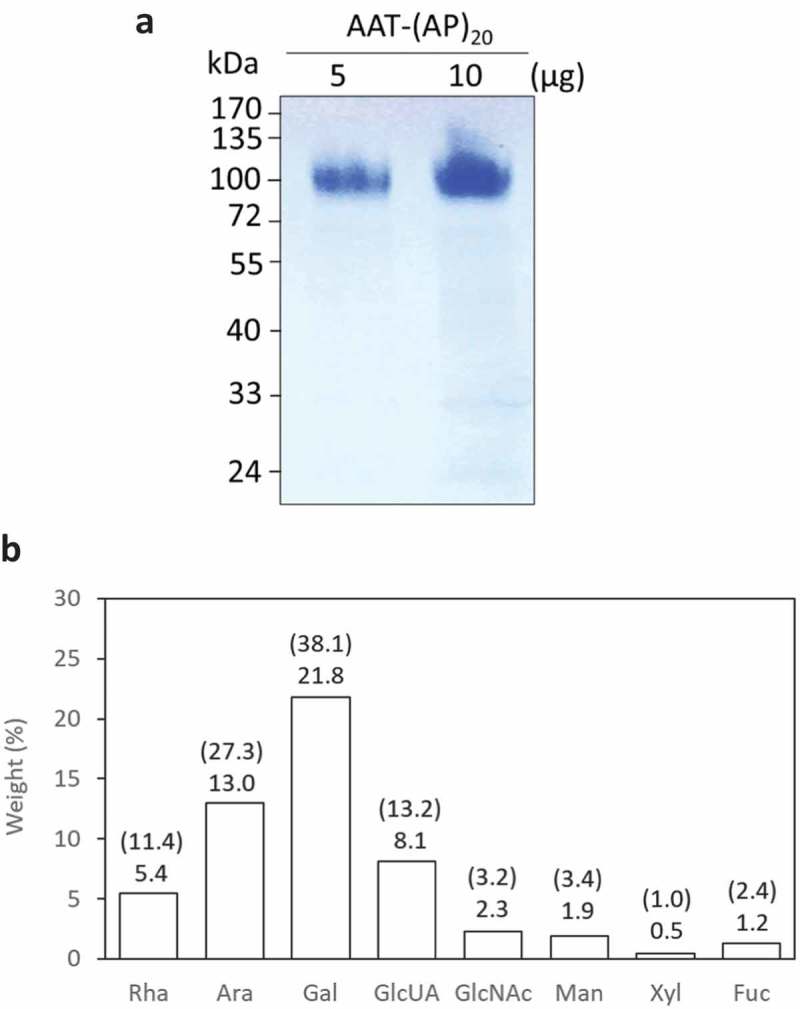
**Biochemical characterization of recombinant AAT-(AP)_20_ fusion proteins.** (a) SDS-PAGE separation of AAT-(AP)_20_ purified from the culture media. (b) Monosaccharide compositions of the AAT-(AP)_20_ fusion proteins. The weight percentage (wt %) of each sugar residue accounts for the total fusion protein is presented and the value indicated on the top of each bar. The number in brackets indicates the molar ratio of each sugar residue in the glycans attached to the AAT-(AP)_20_ polypeptide. Rha, Ara, Gal, and GlcUA are typical sugar residues of Hyp-glycans; GlcNAc, Man, Xyl and Fuc are typical sugar residues of N-glycans.

The secreted AAT and (AP)_20_-tagged AAT products were analyzed by Western blotting with a polyclonal anti-AAT antibody (Figure 4(a)). The AAT with a C-terminal (AP)_20_ tag (AAT-(AP)_20_), migrated as two distinct bands at molecular size of ~110 kDa (upper band) and 40–46 kDa (lower band), respectively. According to calculation based on the polypeptide sequence, the engineered Hyp-*O*-glycosylated (AP)_20_ module would increase the AAT size from 52 kDa to ~106 kDa, which resulted from the (AP)_20_ peptide backbone (3.7 kDa) and the attached 20 Hyp-glycan (~2.5 kDa per glycan as disclosed earlier [26,27]). This indicated the upper band detected in Figure 4(a) corresponded to the AAT-(AP)_20_ fusion glycoprotein. Then, the lower bands (40–46 kDa) must be the truncated AAT domains absent of the (AP)_20_ tag. Right above the lower band occurred a faint band (~52 kDa) that presumably corresponded to the full-length AAT domain as it migrated at the same position as the AAT standard. With the Ni-NTA affinity chromatography, only the intact AAT-(AP)_20_ fusion protein (with a C-terminal 6× His tag) could be purified from culture media (Figure 5(a)). No any lower-band products were recovered by the Ni-NTA columns due to the 6× His tag being cleaved. N-terminal peptide sequencing of the purified protein showed the sequence of ‘EDPQGDAAQKTD…’ (Figure 4(b)), same as that of the native AAT (Supplementary Figure 1), which confirmed that this product (upper band) was the intact AAT-(AP)_20_ fusion protein. It accounted for ~38.5% (w/w) of the total secreted AAT products as estimated by the Western blot densitometry using the Quant-1 software (Bio-Rad, CA).

When the AAT with a N-terminal (AP)_20_ tag was expressed in BY-2 cells, hardly any intact (AP)_20_-AAT fusion protein at ~110 kDa was detected by the anti-AAT Western blot (Figure 4(a)). The major products found were the truncated AAT domains (42–50 kDa) lacking the (AP)_20_ tag, whose molecular sizes were slightly larger than the AAT domains cleaved from the AAT-(AP)_20_ fusion. As in the AAT-(AP)_20_ expression, a faint ~52-kDa band was also observed. N-terminal peptide sequencing detected the sequence of ‘MEDPQGDAAQKTD…’ for the ~52 kDa product and the mixture of ‘TDTSHHDQDH…’ and ‘HDQDHPTFN…’ for the 42–50 kDa products (Figure 4(b)), indicating the (AP)_20_ tag was cleaved at multi-sites from the N-terminus of the fusion protein. Obviously, the ~52 kDa product corresponded to a full-length AAT domain. The first and extra amino acid ‘M’ detected in this domain was introduced while the *AAT-(AP)_20_* gene construct was cloned.

For the expression of AAT control without a (AP)_20_ tag, the products recovered in media existed as multiple truncated polypeptides with molecular sizes ranging from 46 to 50 kDa (Figure 4(a)), similar to those reported earlier in rice cell cultures [14,15]. Again, a faint ~52-kDa band was observed, which could be purified by the Ni-NTA columns and was identified as the full-length AAT based on the peptide sequencing assay (Figure 4(b)). Some of the 46–50 kDa products could also be purified and their N-terminal peptide sequences read almost the same as those of the (AP)_20_-AAT products (Figure 4(b)).

N- and C-terminal truncations of recombinant AAT have been previously reported in plant-produced AAT. In transient expression of AAT in *N. Benthamiana*, both full-length AAT and truncated variants at both termini were found. However, only the truncated version of AAT was detected in intercellular fluids [7]. In inducible expression in rice cells, considerable amounts of recombinant AAT were found to be truncated. In fact, the truncated version of AAT dominated the population of the AAT products secreted into culture media [14–17]. In this study, all the three types of recombinant AAT products secreted from BY-2 cells were subjected to proteolytic degradation (Figure 4). Although the truncation was found to happen at both terminal ends of the expressed AAT, however, the N-terminus was more prone to proteolytic cleavage than the C-terminus, because no intact (AP)_20_-AAT fusion was detected in culture media while ~38.5% secreted AAT-(AP)_20_ fusion protein remaining intact. In addition, the observed smaller sizes of the truncated AAT domains cleaved from the AAT-(AP)_20_ fusion (C-terminal cleavage) than from the (AP)_20_-AAT fusion (N-terminal cleavage) conformed to the frequent cleavage sites of AAT reported earlier: up to 15 amino acids at the N-terminus and up to 41 amino acids at the C- terminus [7].

### *Engineered (AP)_20_ was yp-*o*-glycosylated with arabinogalactan polysaccharides*

This was the first time that ‘Ala-Pro’ motif, rather than ‘Ser-Pro’ motif, was used as a HypGP design to facilitate the secretion of fused protein from cultured plant cells. Our previous studies indicated that extensive Hyp-*O*-glycosylation of the designer HypGP modules was essential in facilitating secretion of the fused protein [21,22,24]. The Hyp-*O*-glycosylation of the engineered (AP)_20_ was then investigated. In this study, only the AAT-(AP)_20_ fusion protein could be purified from culture media at sufficient amount for monosaccharide composition assay (Figure 5(a)). As shown in Figure 5(b), a total of eight sugar residues were detected, which included rhamnose (Rha), arabinose (Ara), galactose (Gal) and glucuronic acid (GlcUA) residues that constitute the Hyp-glycans, and N-acetylglucosamine (GlcNAc), mannose (Man), xylose (Xyl) and fucose (Fuc) residues that are the typical sugar residues of N-glycans. The detected monosaccharide profile demonstrated the AAT-(AP)_20_ fusion protein was both Hyp-*O*-glycosylated and N-glycosylated. The total sugars accounted for 54.2% (w/v) of the fusion protein, of which ~47.1% was contributed by the Hyp-glycans and the rest ~7.1% contributed by the N-glycans, as estimated based on the Hyp-glycan structure elucidated earlier [21,26].

The purified AAT-(AP)_20_ was also tested for its ability to precipitate (*β*-D-galactosyl)_3_-Yariv reagent, which specifically binds AGPs [37]. Like the (SP)_32_-EGFP containing a synthetic (SP)_32_ module, the AAT-(AP)_20_ could react with the Yariv reagent, but not for the AAT control though it bears three N-glycans (Table 1). This confirmed that the glycans attached to the (AP)_20_ module were Type II arabinogalactans typical of classical plant AGPs [26,27]. Compared with the (SP)_32_-EGFP fusion, the AAT-(AP)_20_ generated less amounts of Yariv precipitates, which resulted from the smaller percentage of AGP module present in the AAT-(AP)_20_ ((AP)_20_ accounts for ~51%) than in the (SP)_32_-EGFP ((SP)_32_ accounts for ~77%).

**Table 1.** **Yariv reagent co-precipitation of the AAT-(AP)_20_ fusion protein compared with the (SP)_32_-EGFP.** The data presented represent the mean of three parallel samples ± standard deviation. (SP)_32_-EGFP isolated from BY-2 cell culture medium [24] and AAT standard without the (AP)_20_ module served as positive and negative control, respectively. Twenty-five µg of AAT is roughly equal to the amount of AAT in the AAT-(AP)_20_ fusion glycoprotein.

	Absorbance (420 nm)		
Sample weight (µg)	AAT-(AP)_20_	(SP)_32_-EGFP	AAT (25 µg)
50	0.32 ± 0.01	0.92 ± 0.04	0.01
100	0.72 ± 0.03	1.68 ± 0.06	Not assayed

### BY-2 cell secreted AAT products exhibited biological activity

**Figure 6.**
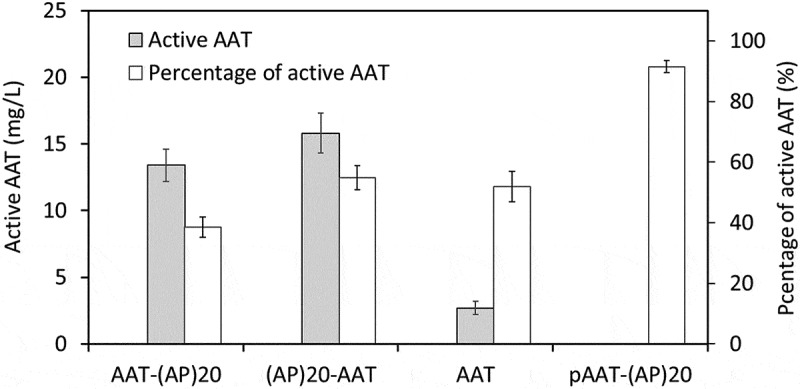
**Biological activities of the recombinant AAT products secreted into BY-2 cell culture media.** The medium samples harvested after 10-day of BY-2 cell culture as well as the purified AAT-(AP)_20_ fusion protein (pAAT-(AP)_20_) were assayed. The percentage of the active AAT accounting for the total AAT products in the samples was then calculated as: active AAT content/total AAT concentration×100%. The error bars represent the standard deviation of three top-expression cell lines for each gene construct (for the medium samples) or three parallel samples (for the pAAT-(AP)_20_ protein).

Biological activity of the secreted AAT products was determined by their ability to inhibit the activity of PPE, a natural target protease. As shown in Figure 6, the active AAT accounted for 38%, 55% and 52% of the total secreted AAT-(AP)_20_, (AP)_20_-AAT and AAT products, respectively. This was similar to the expression of AAT in rice cell cultures, where substantial amounts of secreted recombinant AAT were truncated and lost activity [7,14,15]. For example, [14], reported that only 10% to 20% of the recombinant AAT produced by rice cell culture was functionally active.

Of the two types of (AP)_20_-tagged AAT expressed in BY-2 cell, only the intact AAT-(AP)_20_ fusion protein could be recovered from culture media, and the purified fusion protein exhibited protease inhibitory activity up to 92.5% of the AAT standard. This indicated the engineered (AP)_20_ tag, though heavily *O*-glycosylated with arabinogalactan polysaccharides, did not adversely affect the biological activity of the fused AAT. This was consistent with our previous studies where the engineered (SP)_n_ (n = 5, 10, 20) glycomodule showed limited effects on the bioactivity of two fused therapeutic proteins: interferon α2 and human growth hormone [20,21].

AAT is known to inhibit serine proteases. It contains an exposed and mobile reactive center loop (RCL) at C-terminus with a methionine (^358^M) residue acting as bait for target proteinases [38]. Since the active site of the AAT is located near the C-terminal end, proteolytic cleavage at C-terminal of AAT, particularly occurring within RCL, destroys the RCL structure and thereby renders AAT inactive [7]. For the recombinant AAT secreted from rice cell cultures, the AAT having molecular sizes around 48–50 kDa was found to remain biologically active, but those with molecular size around 42–44 kDa were inactive [15]. In this study, most of the BY-2 cell secreted AAT products were truncated at either terminal ends. Not only the (AP)_20_ tag was cleaved from the fusion proteins, but also the cleaved AAT domains were truncated. It seemed that the AAT domains cleaved from the AAT-(AP)_20_ fusion lost more amino acids, most possibly at the C-terminal end, than those from the (AP)_20_-AAT fusion or the AAT control, because the former migrated to a lower position (40–46 kDa) than the latter (42–50 kDa) on the SDS-PAGE (Figure 4(a)). This also implied that the C-terminal (AP)_20_ tag might facilitate the proteolytic degradation at sites preceding RCL. Due to loss of the active site (RCL) at the C-terminal end, most of the AAT domains cleaved from the AAT-(AP)_20_ became inactive. This was reflected by the finding that less active AAT populations in the AAT-(AP)_20_ cell culture media (38%) than in the (AP)_20_-AAT or AAT media (52–55%) (Figure 6). In fact, the active AAT population in the AAT-(AP)_20_ media was largely contributed by the intact AAT-(AP)_20_ fusion protein that retained a high biological activity (92.5%). This also indicated that many truncated AAT domains from the (AP)_20_-AAT or AAT still kept the RCL site at their C-terminal ends and thus remained biologically active.

## Conclusions

The present study is the first report on the expression of human AAT in tobacco BY-2 cells. Engineering a designer HypGP tag composed of the (AP)_20_ motif at either N- or C-terminal end of AAT substantially increased the secreted protein yields from 5.2 mg/L up to 34.7 mg/L (a 6.7-fold increase). The secreted recombinant AAT products, including the intact AAT-(AP)_20_ fusion protein and some of the truncated AAT domains, exhibited desired protease inhibitory activity. Our results demonstrated that the HypGP engineering technology could be applied to the expression of a relatively large protein-AAT in BY-2 cell to substantially increase the secreted yields.

## Highlights

- Synthetic (AP)_20_ module is extensively Hyp-O-glycosylated in BY-2 cell
- (AP)_20_ module facilitates extracellular secretion of fused α1-antitrypsin
- BY-2 secreted α1-antitrypsin are subjected to proteolytic processing
- (AP)_20_–tagged α1-antitrypsin exhibits biological activity in inhibiting elastase
