# Supplementary material for: Enhanced secretion of human α1-antitrypsin expressed with a novel glycosylation module in tobacco BY-2 cell culture
Source: Bioengineered. 2019 Apr 17;10(1):87–97. doi: 10.1080/21655979.2019.1604037 (PMC6527068; doi:10.1080/21655979.2019.1604037)
Supplement: Supplemental Material [file kbie-10-01-1604037-s001.docx]

**Enhanced Secretion of Human α1-Antitrypsin Expressed with a Novel Glycosylation Module in Tobacco BY-2 Cell Culture**

Ningning Zhang^1^, Tristen Wright^2^, Paige Caraway^2^, Jianfeng Xu^1,3,^**^*^**

^1^Arkansas Biosciences Institute, ^2^Department of Biological Sciences, ^3^College of Agriculture, Arkansas State University, Jonesboro, AR 72401, USA

**Supplementary Table 1. Primers used in this study**

| Primer name | Sequence | Restriction sites |
| --- | --- | --- |
| AAT-F1 | 5’-GCTCCTGCCCCCATGGAGGATCCCCAGGGAGATGC-3’ | *Nco*I |
| AAT-F2 | 5’-GCACAAACAACCCGGGAGGATCCCCAGGGAGATGC-3’ | *Xma*I |
| AAT-R | 5’-CCGCTTTACTTGTACATTAGTGATGATGGTGGTGATGT TTTTGGGTGGGATTCACCAC-3’ | *BsrG*I |
| SS-F | 5’-ATGGGAAAAATGGCTTCTC-3’ |  |
| SS-R | 5’-GAGCTCCACCGCGGTGGCG-3’ | *Sac*I |


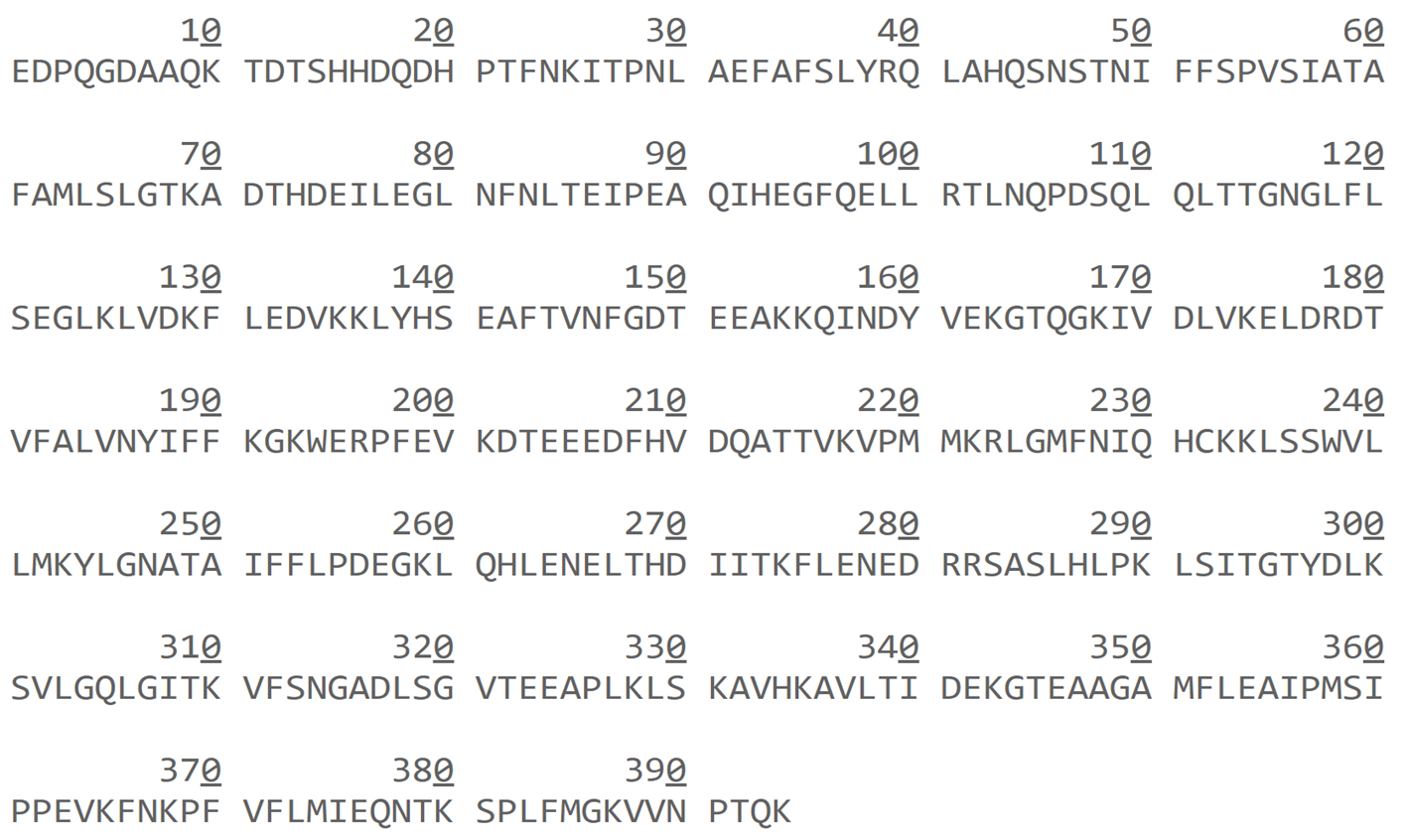


**Supplementary Fig. 1 Amino acid sequence of α1-Antitrypsin (AAT).** Theoretical molecular weight of AAT is 44.3 kDa (non-glycosylated). Apparent molecular size of matured AAT is 52 kDa (with three N-linked glycans).
